# Mapping QTL for Seed Germinability under Low Temperature Using a New High-Density Genetic Map of Rice

**DOI:** 10.3389/fpls.2017.01223

**Published:** 2017-07-12

**Authors:** Ningfei Jiang, Shilai Shi, Huan Shi, Hira Khanzada, Ghulam M. Wassan, Changlan Zhu, Xiaosong Peng, Qiuying Yu, Xiaorong Chen, Xiaopeng He, Junru Fu, Lifang Hu, Jie Xu, Linjuan Ouyang, Xiaotang Sun, Dahu Zhou, Haohua He, Jianmin Bian

**Affiliations:** ^1^Key Laboratory of Crop Physiology, Ecology and Genetic Breeding, Ministry of Education, Jiangxi Agricultural University Nanchang, China; ^2^College of Agronomy, Jiangxi Agricultural University Nanchang, China; ^3^Southern Regional Collaborative Innovation Center for Grain and Oil Crops in China Changsha, China

**Keywords:** rice, low temperature, seed germinability, high-density genetic map, fine mapping

## Abstract

Mapping major quantitative trait loci (QTL) responsible for rice seed germinability under low temperature (GULT) can provide valuable genetic source for improving cold tolerance in rice breeding. In this study, 124 rice backcross recombinant inbred lines (BRILs) derived from a cross *indica* cv. Changhui 891 and *japonica* cv. 02428 were genotyped through re-sequencing technology. A bin map was generated which includes 3057 bins covering distance of 1266.5 cM with an average of 0.41 cM between markers. On the basis of newly constructed high-density genetic map, six QTL were detected ranging from 40 to 140 kb on Nipponbare genome. Among these, two QTL *qCGR8* and *qGRR11* alleles shared by 02428 could increase GULT and seed germination recovery rate after cold stress, respectively. However, *qNGR1* and *qNGR4* may be two major QTL affecting *indica* Changhui 891germination under normal condition. QTL *qGRR1* and *qGRR8* affected the seed germination recovery rate after cold stress and the alleles with increasing effects were shared by the Changhui 891 could improve seed germination rate after cold stress dramatically. These QTL could be a highly valuable genetic factors for cold tolerance improvement in rice lines. Moreover, the BRILs developed in this study will serve as an appropriate choice for mapping and studying genetic basis of rice complex traits.

## Introduction

Low temperature germination is a major determinant for stable establishing of the cultivated rice in tropical or subtropical area where optimum temperatures for rice growth during germination and seedling stages are from 25 to 35°C (Fujino et al., 2004; Xu et al., 2015). The low temperature existing during seed germination stage results poor plants survival rate and alternatively cause reductions in the final yield. Cold tolerance improvement at germination stage is therefore necessary for yield stability and an increase in crop productivity (Fujino et al., 2008). Moreover, paddy field with direct seeding become one of the choice with low labor and cost to overcome the damage of low temperature in rice cultivars (Mao et al., 2015). Improving cold tolerance in rice varieties with conventional breeding methods is labor-and time consuming as cold tolerance is complicated and controlled by quantitative trait loci (QTL). Thus, better understanding of the complexity of QTL underlying cold tolerance at the seed germination stage is estimated to accelerate the progress of cold-tolerant cultivars development by using molecular assisted breeding technology (Koseki et al., 2010).

Rice has been divided into two main subspecies, *indica* and *japonica*. In general, *indica* rice cultivars are cold sensitive and are distributed mainly in tropical and subtropical regions, while *japonica* cultivars have tolerance to low temperatures and well adapt to temperate climates or higher altitudes (Oka, 1958; Mao et al., 2015). In other words, optimization of these two subspecies become necessary to improve cold-tolerant of rice cultivars.

To find out the genetic basis of cold at germination stage for *indica* and *japonica*, several independent studies have been reported and some low-temperature germinability related QTL have been identified in different populations (Miura et al., 2001; Zhang et al., 2005; Jiang et al., 2006; Ji et al., 2008; Wang Z. et al., 2011; Li et al., 2013; Satoh et al., 2016). For example, Miura et al. (2001) detected five low temperature germinability related QTL on chromosomes 2, 4, 5, and 11. Zhang et al. (2005) identified 34 QTL associated with seedling vigor traits (including germination rate, shoot length, root length, and dry weight) by using three temperature regimes (15, 20, and 25°C) (Borjas et al., 2016). Jiang et al. (2006) reported eleven putative QTL for low temperature germination ability on chromosomes 3–5, 7, 9–11 in an *indica* × *japonica* cross. Ji et al. (2008) carried out a low-temperature germinability genetic analysis and a total of 11 QTL on chromosomes 2, 5, 7, 8, 11, and 12 were detected. Wang Z. et al. (2011) studied cold tolerance at germination stage in a *japonica* × *indica* cross, two minor QTL responsible for cold tolerance at germination were observed. Li et al. (2013) found three QTL (*qLTG-7, qLTG-9*, and *qLTG-12*) associated with low-temperature germination, and *qLTG-9* was fine mapped to a 72.3-kb region in chromosome 9. Among these QTL, only one QTL (*qLTG3-1*) has been cloned (Fujino et al., 2008). This is because all these low-temperature germinability related QTL were identified based on traditional markers such as SSRs (simple sequence repeats), which were always sparsely distributed on rice 12 chromosomes; thus it is very difficult to get precise and complete information for these low-temperature germinability QTL (Mao et al., 2015). These uncertain results hindered the mapping of these QTL and limit our understanding of the cold-tolerant presence for *indica* and *japonica*. Therefore, the development of new type makers is necessary to facilitate fine mapping and cloning of QTL.

Recently, advances in whole-genome sequencing approach have provided an effective platform for direct identification of million of single nucleotide polymorphisms (SNPs) across the whole genome. The adjacent SNPs with the same genotype in an internal are combined into bins via sliding-window approach (Huang et al., 2009); these bins could demarcate recombination events across the whole population and be used as an effective type of genetic markers for QTL analysis (Wang L. et al., 2011). The high density map developed by the bin-markers has successfully accelerated the genetic studies for quantitative traits in a variety of species (Xie et al., 2010; Xu et al., 2010; Poland et al., 2012; Byrne et al., 2013; Sonah et al., 2013; Wang et al., 2015).

In this study, high-resolution QTL mapping have been reported through sequencing-based genotyping of 124 rice backcross recombinant inbred lines (BRILs). The population was generated from the crossing *Oryza sativa* spp. *indica* Changhui 891 with *Oryza sativa* ssp. *japonica* 02428. A total of six QTL within relatively small genomic regions were identified for rice germinability under low temperature (GULT). The high-resolution QTL mapping method thus greatly improved the precision and resolution of QTL mapping, meanwhile, the QTL within relatively small genomic regions for rice germination will facilitate the development of novel cold-tolerant cultivars using molecular breeding strategies (Koseki et al., 2010).

## Materials and Methods

### Mapping Population

Two rice (*Oryza sativa* L.) varieties; Changhui 891, an excellent *indica* restorer line in south China, and 02428, a leading *japonica* wide compatibility variety, were used to develop this mapping population. A BRILs (BC_1_F_6_) population consisting of 124 individuals derived from Changhui 891/02428//02428 was developed for subsequently analysis. The population was developed in the experimental field at Jiangxi Agricultural University in Nanchang, Jiangxi Province, and Linwang, Hainan Province. After continuous of six generations of self-fertilization, the genomic DNA from each line of the BC_1_F_6_ BRILs was extracted and subjected for genotyping.

### Evaluating Germinability under Low Temperature

The seeds with high-quality were selected by removal of shriveled and unfilled seeds. The seeds were surface-sterilized by dipping 15 min in a 10% solution of sodium hypochlorite and were rinsed for three times with fresh clean tap water (Satoh et al., 2016). Each sample with 30 seeds was placed in a Petri dish containing 5 ml of tap water and incubated at 15°C for 1 week in a growth chamber. Tap water was added every day according to requirement to avoid desiccation, germinated seeds were counted on the seventh day.

The germination potential of seeds that did not germinate at low temperature were evaluated by further incubation of these seeds at 25°C for 24 h and the germinated seeds were counted on the eighth day. The germination test was repeated two times for each line and the germination rate (%) was calculated as: Germination rate (%) = (number of germinated seeds during the test period/total number of germinated seeds) × 100; Seed germination recovery rate = [(number of germinated seeds on eighth day - number of germinated seeds on seventh day)/number of total germinated seeds] × 100.

### Genotyping, Linkage Map Construction

Healthy and young fresh leaves were collected from 124 BRILs along with their parental lines and subjected to DNA extraction using cetyltrimethylammonium bromide (CTAB) method with minor modifications. Each DNA was cut by an enzyme Ecor I and the adapters were ligated to barcode the DNA of each line. Size of each sample was selected by gel electrophoresis and DNA fragments were consisted of 300–500 bp in length. Finally, the libraries were enriched by PCR amplification and sequenced was performed using Illumina HiSeq 4000 instrument using paired-end reads (100 bp). The raw reads were then filtered and sorted according to indices and the high-quality SNPs between parents were termed by alignment with Nipponbare reference genome^1^.

Due to the high linkage disequilibrium, high-density genetic map of populations often contain many redundant markers that provides no any new information. Otherwise, a small number of genotypes are falsely termed due to sequencing error. To overcome these issues, a modified sliding-window approach was recommended (Huang et al., 2009). According to the method of Chen et al. (2014), consecutive 40-kb intervals that lack a recombination event in the population were combined into bins via sliding-window approach (Huang et al., 2009); these bins could demarcate recombination events across the whole population and be used as a genetic marker for linkage map construction using IciMapping v4.1 Mapping software^2^.

### QTL Analysis in BRIL Population

To explore the gene expression for seed germination for the BRIL population, QTL IciMapping v4.1 software was used for QTL analysis, threshold LOD of 2.5 was applied (Bian et al., 2015). QTL nomenclature was followed by the method of McCouch et al. (2008).

## Results

### Sequencing and Genotyping

**FIGURE 1:**
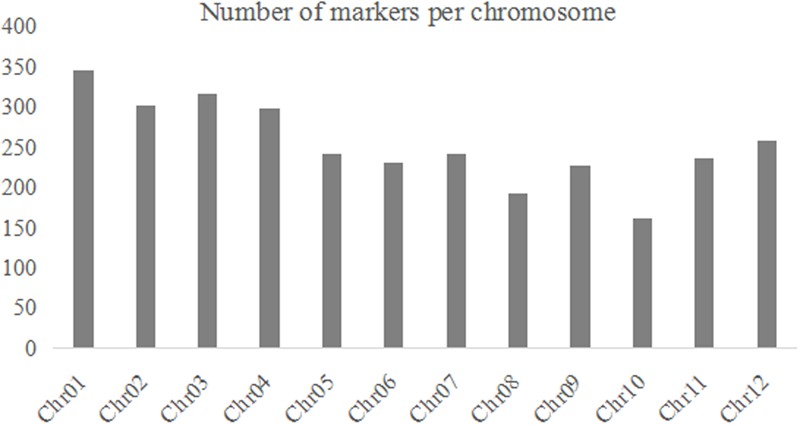
The number of bin markers for each chromosome in the BRIL population.

For each BRIL individual, the reads of the 100-bp sequences were sort out on indices basis. A total of ∼890.09 million reads with average of ∼7.18 million reads per BRIL individual were generated (Supplementary Table S1). The ratio of Q20 for each sample was above 90%, thus the quality of the data is very high and meet the requirements for further analysis. The sequencing reads were then aligned to reference genome using SOAP2 software (Li et al., 2009b) and SNPs were identified with the SOAPsnp software package (Li et al., 2009a). The SNP numbers of parental lines 02428 and Changhui 891 are 22,358 and 85,605 as compared to the Nipponbare rice reference. Finally, a total of 70,480 high-quality SNPs were detected in two parents were selected (**Figure 1**). According to the method as described above, a total of 3057 bin markers were obtained to construct the recombination map.

### Linkage Map of Recombination Bins

**FIGURE 2:**
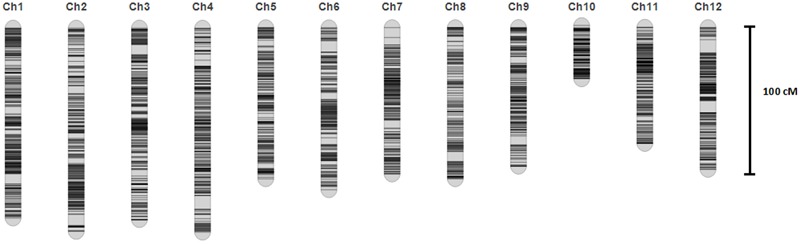
The high density map of the BRIL population.

**FIGURE 3:**
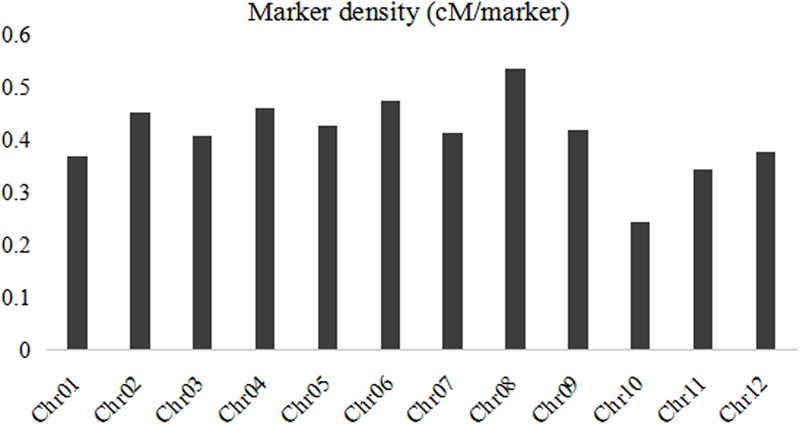
The bin marker density for each chromosome in the BRIL population.

A linkage map was developed using the 3057 recombination bins which were generated from whole-genome re-sequencing of the 124 BRILs. Total genetic distance of linkage map was 1266.5 cM with an average interval of 0.41 cM between adjacent bins (**Supplementary Data Sheet 1** and **Figure 2**). The genetic distance between adjacent bins was ranging from 0 to 9.07 cM. The 12 rice linkage groups varied in the number of markers and marker density (**Figures 2, 3**). Total marker density (0.24 cM/marker) was highest in linkage group on chromosome 8. The lowest marker density (0.54 cM/marker) was in linkage group on chromosome 10.

### Phenotypic Performance of BRIL Population for Cold Tolerance

**FIGURE 4:**
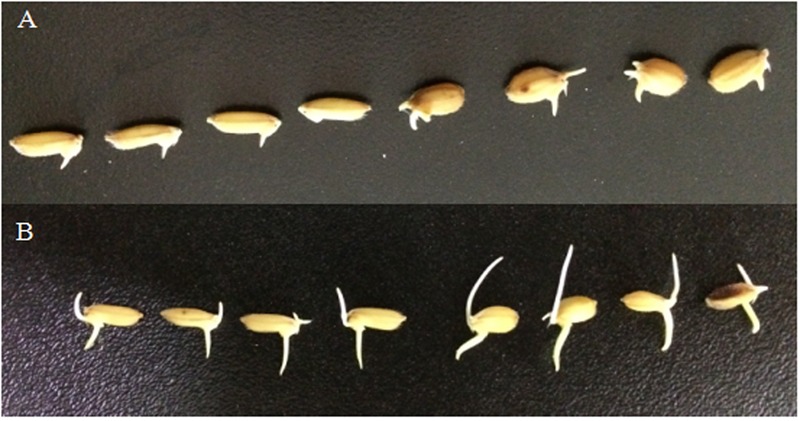
The germination of the two parents: Changhui 891 (left) and 02428 (right) on seventh day **(A)** and eighth day **(B)**.

Overall, 41.7% of the parental line 02428 seeds were germinated at 15°C at 7 days after sowing, whereas most of the Changhui 891 seeds had not germinated (**Table 1** and **Figure 4**). While after incubation at 25°C for 24 h after the germination test at low temperature, the germinate rate of 02428 line was 75.0% while Changhui 891 had germinated rate of 70.0%. The seed germinate recovery rate of Changhui 891 (68.3%) is higher than that of 02428 (33.3%) (**Figure 4**).

**Table 1:** Performance of parents and BRIL population for seed germination under cold stress.

Traits	02428	Changhui 891	BRIL population	
	Mean ± SD^a^	Mean ± SD	Range	Mean
Seed germination rate on seventh day under cold stress (%)	41.7 ± 2.4	1.7 ± 2.4	0.0–73.3	21.7
Seed germination rate on eighth day under cold stress (%)	75.0 ± 2.4	70.0 ± 4.7	18.3–96.7	76.4
Seed germination recovery rate (%)	33.3 ± 4.7	68.3 ± 7.1	6.7–96.7	54.7

*^a^SD, standard deviation.*

The average number of seed germination % per each BRIL individual was 21.7% ranging from 0 to 73.3% under 15°C at 7 days after sowing (**Table 1**). After incubation at 25°C for 24 h of the germination test at low temperature, the average germinate rate was 76.4% with a range of 18.3 to 96.7%, and in the end, seed germinate recovery rate was 54.7% with a range of 6.7 to 96.7% in the BRIL population.

### QTL Analysis for Cold Germination in BRIL Population

**FIGURE 5:**
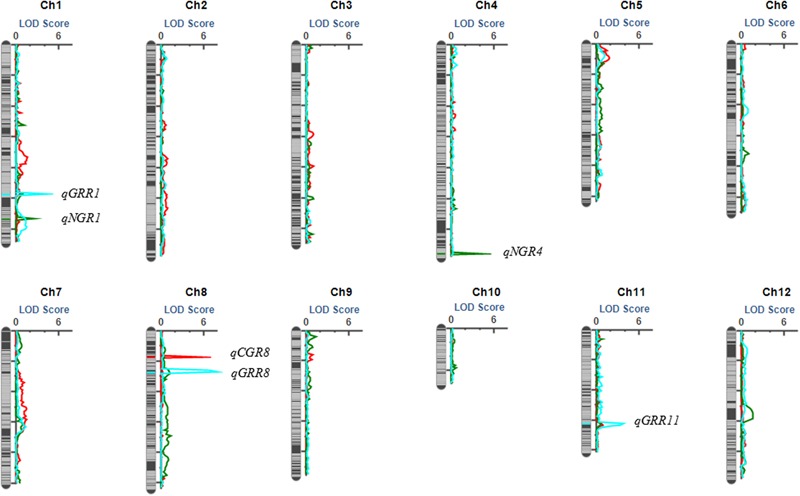
The QTL position on the high density map of the BRIL population.

In total, six QTL were identified by interval mapping with IciMapping v4.1 software using high-density map, One QTL for seed germination on seventh day, two QTL for seed germination on eighth day and three QTL for seed germination rate recovery after cold stress were identified in BRIL population (**Table 2** and **Figure 5**).

**Table 2:** Putative QTL associated with seed germination under cold stress in the BRIL population in rice.

Traits	QTL	Chr.^a^	Left Marker (physical position)	Right Marker (physical position)	LOD^b^	ADD^c^	PVE (%)^d^
Seventh day under cold stress	*qCGR8*	8	bin 8-28 (2,820,000)	bin 8-29 (2,860,000)	5.74	0.08	20.71
Eighth day after cold stress	*qNGR1*	1	bin 1-314 (41,100,000)	bin 1-315 (41,140,000)	2.52	-0.05	6.31
	*qNGR4*	4	bin 4-289 (35,060,000)	bin 4-290 (35,100,000)	4.42	-0.05	10.43
Seed germination recovery rate	*qGRR1*	1	bin 1-218 (29,140,000)	bin 1-219 (29,280,000)	4.33	-0.07	11.93
	*qGRR8*	8	bin 8-53 (5,500,000)	bin 8-54 (5,620,000)	7.84	-0.1	23.33
	*qGRR11*	11	bin 11-198 (25,500,000)	bin 11-199 (25,540,000)	3.78	0.09	11.08

*^a^Chr., chromosome.*

*^b^LOD, logarithm of odds.*

*^c^ADD, Additive effect; a positive value indicates superiority of the *japonica* 02428.*

*^d^PVE (%), percent of variance explained (%).*

#### QTL Mapping for Germination on Seventh Day under Cold Stress

Only one QTL (*qCGR8*) for seed germination on seventh day was detected with phenotypic variation explained (PVE) of 20.71% and mapped on a region between bin 8-28 and bin 8-29 on chromosome 8. The genetic distance was 0.2 cM and the physical distance was 40 kb in Nipponbare genome. The allele with increasing effects on the seed germination was shared by *japonica* 02428.

#### QTL Mapping for Germination on Eighth Day under Cold Stress

Two QTL for seed germination on eighth day were detected. QTL (*qNGR1*) was harbored on chromosome 1 on a region between bin 1-314 and bin 1-315 and covered the genetic distance was about 1.15 cM, and physical distance was about 40 kb in Nipponbare genome. QTL *qNGR4* was identified on chromosome 4 and covered genetic distance of about 0.2 cM between bin 4-289 and bin 4-290, and the physical distance was about 40 kb in Nipponbare genome. The alleles with increasing effects for seed germination rate were shared by *indica* Changhui 891 line.

#### QTL Mapping for Seed Germination Recovery Rate

Among the three QTL detected for seed germination rate recovery after cold stress, QTL (*qGRR8*) was mapped on chromosome 8 on the region of bin 8-53 and bin 8-54. It contributed the largest PVE value (23.33%). The genetic distance was 1.96 cM, and a physical distance was about 120 kb in Nipponbare genome. The QTL (*qGRR1*) is located between bin 1-218 and bin1-219 on chromosome 1, the genetic distance covered is 0.2 cM, and a physical distance of about 140 kb to corresponding genome. While QTL (*qGRR11*) is detected on chromosome 11 between bin 11-198 and bin11-199, the genetic distance was about 0.62 cM, and a physical distance of about 40 kb in Nipponbare genome. The alleles with the increasing additive effects on seed germination recovery rate after cold stress for QTL *qGRR1* and *qGRR8* were from Changhui 891 while for QTL *qGRR11* were shared by parental line 02428.

## Discussion

### Development of High Density Bin-map Using BRIL Population Enables the Genetic Analysis of Quantitative Traits in Rice

An appropriate mapping population is effective in QTL analysis and gene cloning. Due to genetic background noise, the standard segregating populations cannot give an precise information (the location and the size) for each individual QTL, particularly small effect QTL. Near-isogenic lines (NILs) (CSSLs) and chromosome segment substitution lines can overcome this issue and therefore ideal for QTL detection (Kubo et al., 1999; Bian et al., 2010), but they are time consuming and labor-intensive which limited the acceleration of gene cloning. BRILs being a permanent population are easy to develop compare to CSSLs and NILs. Moreover, because of the high percentage of recurrent parent genome, it can be rapidly crossed with the recurrent parent to construct NILs for the target QTL cloning. Therefore, it become necessary to develop BRIL population for the genetic analysis of quantitative traits in rice (Wang B. et al., 2011; Hosseini et al., 2012). In this study, a BRIL population consisting of 124 individuals derived from Changhui 891/02428//02428 was developed, hence we assume this population will serve as better choice for mapping and studying important quantitative traits of rice.

Quantitative trait loci mapping resolution depended on the marker density and the size of confidence interval of QTL (Visscher et al., 1996; Da et al., 2000). Generally speaking, a gain of information by more markers density resulted in smaller intervals, therefore, the application of additional markers is an effective way to increase QTL mapping resolution (Liu et al., 2008). The traditional markers such as SSRs and restriction fragment length polymorphism (RFLP) are not suitable when their linkage distance are zero and cannot meet the requirements of the high QTL mapping resolution. Development of SNP markers by next-generation sequencing technologies will increase the feasibility to increase QTL mapping resolution. In this study, an ultra-high density bin-map for the BRIL population was constructed through re-sequence strategy and used for QTL mapping in rice for seed germination. Compared with previous QTL associated with rice seed germination, the QTL interval size in this study was significantly narrow and ranged from 40 to 140 kb in Nipponbare genome. These results confirmed that genotyping by sequencing has significantly increased the QTL mapping resolution compared with traditional markers.

### Relationship of Detected QTL and Previously Identified Genes

The position of *qCGR8* on chromosome 8 is near to the major QTL *qCTS8.1* which is responsible for increasing seedling cold tolerance in *japonica* rice variety Daugandao (Wang Z. et al., 2011). These varieties may thus share the QTL for cold tolerance. In this study, *qCGR8* was identified on a region about 0.2 cM, and the physical distance was about 40 kb. Six candidate genes were predicted, which provide a good clue for the cold tolerance gene isolation (**Table 3**). Otherwise, chromosome 11 locus *qGRR11* maps to a similar location as does by *qLTG11-2* (Jiang et al., 2006) and *qLTG11-2* (Wang Z. et al., 2011). It has been noticed that the region (marker by bin 11-198–bin 11-199) may play an important role in seedling germination under low temperature (Satoh et al., 2016). Identification of the gene from different varieties allow us to understand the roles of this region in seedling germination (Satoh et al., 2016) but the accurate location for this QTL is still not clear. Luckily, *qGRR11* was mapped between bin11-198 and bin11-199 on chromosome 11 in this study covering the genetic distance of 0.62 cM, corresponding to a physical distance of about 40 kb. Six candidate genes were predicted in this region (**Table 3**), it would be only a matter of time before the isolation of *qNGR11*. Four new QTL; *qNGR1, qNGR4, qGRR1*, and *qGRR8* are newly detected QTL and never reported before, these all QTL were located on small genomic regions (40–140 kb). This result contributes to the fine mapping of these QTL and provides a foundation for understanding the mechanism of rice germination and seedling growth under low-temperature conditions (Li et al., 2013). Moreover, the BRILs harboring these QTL will serve as an excellent materials and enable us to map and study genetic basis of GULT.

**Table 3:** The candidate genes for QTL *qCGR8* and *qGRR11.*

QTL	Locus name	CDS coordinates	Gene product name
*qCGR8*	LOC_Os08g05360.1	2822433 – 2830063	Transposon protein, putative, unclassified, expressed
	LOC_Os08g05370.1	2835215 – 2833168	Conserved hypothetical protein
	LOC_Os08g05380.1	2838127 – 2838734	Expressed protein
	LOC_Os08g05390.1	2842969 – 2839931	Transposon protein, putative, CACTA, En/Spm sub-class, expressed
	LOC_Os08g05400.1	2847336 – 2845858	Transposon protein, putative, CACTA, En/Spm sub-class, expressed
	LOC_Os08g05410.1	2852459 – 2857610	Expressed protein
*qGRR11*	LOC_Os11g42370.1	25501586 – 25503169	Transferase family protein, putative, expressed
	LOC_Os11g42380.1	25504028 – 25503455	Expressed protein
	LOC_Os11g42390.1	25509587 – 25515718	OsSCP64 – Putative serine carboxypeptidase homolog, expressed
	LOC_Os11g42400.1	25519158 – 25519909	Expressed protein
	LOC_Os11g42410.1	25526265 – 25522647	Expressed protein
	LOC_Os11g42420.1	25528232 – 25537466	Nuclear pore protein 84/107 containing protein, expressed

### The QTL Identified Will Be Useful for Molecular Breeding of Cold-Tolerant Rice

Vigorous GULT is an important agricultural trait for direct seeding in rice especially at high altitudes in subtropical and tropical regions, and in areas with cold irrigated water (Fujino et al., 2004; Li et al., 2013; Mao et al., 2015; Satoh et al., 2016). Generally, GULT of *japonica* varieties was higher than that of *indica* varieties, cold resistant genes/QTL from *japonica* rice have been used as gene resource to improve the cold resistance in *indica* rice (Mao et al., 2015). However, only few of these QTL are considered appropriate for improving cold tolerance in *indica* because of the ambiguous chromosome position. In present study, six QTL ranged from 40 to 140 kb in Nipponbare genome were fine-mapped, further small genomic region can be transferred directly by molecular assisted selection (MAS). Among these, two QTL, *qCGR8* and *qGRR11* whose alleles from 02428 could increase GULT and seed germination recovery rate after cold stress, respectively. These QTL will be available in cold-tolerant rice breeding programs especially for cold-tolerant *indica* rice breeding program. For *qNGR1* and *qNGR4*, the increasing effect of alleles were detected from Changhui 891 for seed germination and may be two major QTL from Changhui 891 affecting germination under normal condition. However, two QTL *qGRR1* and *qGRR8* affecting the seed germination recovery rate after cold stress, the alleles from Changhui 891 could improve seed germination rate dramatically after cold stress. This also suggest that alleles shared by Changhui 891 for QTL *qGRR1* and *qGRR8* would be a highly valuable genetic factors for improving cold tolerant in rice lines. In other words, the rice lines harboring Changhui 891 alleles for *qGRR1* and *qGRR8* could significantly improve the seed germinability even after a period of cold stress.

## Author Contributions

JB and HH designed the research work, annotated the data and drafted the manuscript. NJ, HS, SS, CZ, XP, and QY performed the experiments. XC, XH, JF, LH, LO, JX, XS, and DZ developed the population. HK and GW revised the manuscript for the language.

## Conflict of Interest Statement

The authors declare that the research was conducted in the absence of any commercial or financial relationships that could be construed as a potential conflict of interest.

## Footnotes

1 ftp://ftp.ensemblgenomes.org/pub/release-21/plants/fasta/oryza_sativa/dna/

2 http://www.isbreeding.net/software/?type=detail&id=18

## Supplementary Material

DATA SHEET 1 **The bin marker information of the high density map**.
