# Supplementary material for: Mapping QTL for Seed Germinability under Low Temperature Using a New High-Density Genetic Map of Rice
Source: Front Plant Sci. 2017 Jul 12;8:1223. doi: 10.3389/fpls.2017.01223 (PMC5506081; doi:10.3389/fpls.2017.01223)
Supplement: Supplementary file 2 [file Data_Sheet_2.DOC]

**Table S1** The clean reads related data of parents and 124 BRILs

| Smple ID | Sample name | Read number (M) | Base number (Mb) | GC (%) | Q20 (%) | Q30 (%) |
| --- | --- | --- | --- | --- | --- | --- |
| 1 | BQ1 | 8.99 | 871.57 | 40.32 | 92.41 | 81.73 |
| 2 | BQ2 | 6.05 | 586.41 | 39.61 | 92.71 | 82.18 |
| 3 | BQ3 | 6.74 | 660.69 | 40.12 | 92.49 | 81.89 |
| 4 | BQ4 | 5.03 | 490.16 | 40.16 | 92.73 | 82.32 |
| 5 | BQ5 | 7.68 | 748.38 | 40.66 | 93.35 | 83.67 |
| 6 | BQ6 | 5.31 | 512.08 | 40.96 | 91.76 | 80.39 |
| 8 | BQ8 | 8.43 | 821.6 | 40.63 | 94.16 | 86.76 |
| 9 | BQ9 | 6.45 | 619.47 | 40.66 | 92.64 | 82.52 |
| 11 | BQ11 | 6.02 | 581.19 | 39.99 | 91.06 | 79.05 |
| 12 | BQ12 | 8.52 | 818.25 | 40.59 | 93.01 | 83.3 |
| 13 | BQ13 | 6.59 | 632.73 | 40 | 92.14 | 81.42 |
| 15 | BQ15 | 4.67 | 448.3 | 41.06 | 93.13 | 83.29 |
| 18 | BQ18 | 7.29 | 706.66 | 41.67 | 93.17 | 83.41 |
| 19 | BQ19 | 6.08 | 589.3 | 41.53 | 93.44 | 83.81 |
| 21 | BQ21 | 6.95 | 680.63 | 42.02 | 92.93 | 83.02 |
| 22 | BQ22 | 7.43 | 721.03 | 40.53 | 93.63 | 84.17 |
| 23 | BQ23 | 8.64 | 842.82 | 41.42 | 93.27 | 83.6 |
| 25 | BQ25 | 7.46 | 727.2 | 41.87 | 93.58 | 84.14 |
| 26 | BQ26 | 5.86 | 574.19 | 39.99 | 93.52 | 84.05 |
| 27 | BQ27 | 10.24 | 988.58 | 41.69 | 91.94 | 80.78 |
| 28 | BQ28 | 9.61 | 922.87 | 42.05 | 92.67 | 82.68 |
| 29 | BQ29 | 6.6 | 633.48 | 41.5 | 93.21 | 83.54 |
| 30 | BQ30 | 6.78 | 657.76 | 40.76 | 92.2 | 82.03 |
| 31 | BQ31 | 6.46 | 627.01 | 41.04 | 92.25 | 82.06 |
| 32 | BQ32 | 8.12 | 795.84 | 41.08 | 92.26 | 82.16 |
| 34 | BQ34 | 5.41 | 527.29 | 40.7 | 92.27 | 82.2 |
| 40 | BQ40 | 7.77 | 757.8 | 40.68 | 92.5 | 82.57 |
| 41 | BQ41 | 6.09 | 587.83 | 40.62 | 90.89 | 79.43 |
| 42 | BQ42 | 6.24 | 598.58 | 40.64 | 92.05 | 81.8 |
| 44 | BQ44 | 5.03 | 482.73 | 40.98 | 92.17 | 81.96 |
| 45 | BQ45 | 6.93 | 672.61 | 40.63 | 93.17 | 83.78 |
| 46 | BQ46 | 7.31 | 709.52 | 40.51 | 93.3 | 83.93 |
| 47 | BQ47 | 6.77 | 659.86 | 39.82 | 93.97 | 84.86 |
| 48 | BQ48 | 7.14 | 688.92 | 40.52 | 92.43 | 81.65 |
| 50 | BQ50 | 7.41 | 725.86 | 40.56 | 93.19 | 83.84 |
| 51 | BQ51 | 5.92 | 568.76 | 40.43 | 93.08 | 83.39 |
| 52 | BQ52 | 6.27 | 611.63 | 40.55 | 93.15 | 83.77 |
| 53 | BQ53 | 7.11 | 682.25 | 40.05 | 93.75 | 84.48 |
| 54 | BQ54 | 7.24 | 702.1 | 39.89 | 92.6 | 82.31 |
| 55 | BQ55 | 8.75 | 849.13 | 39.88 | 92.75 | 82.49 |
| 56 | BQ56 | 8.7 | 848.64 | 40.5 | 93.36 | 84.14 |
| 57 | BQ57 | 7.95 | 766.89 | 40.47 | 91.93 | 81.19 |
| 62 | BQ62 | 7.53 | 723.25 | 40.56 | 92.96 | 83.43 |
| 65 | BQ65 | 6.95 | 667.04 | 40.57 | 93.18 | 83.74 |
| 66 | BQ66 | 5.83 | 565.21 | 40.31 | 91.85 | 80.89 |
| 67 | BQ67 | 4.93 | 483.01 | 40.65 | 94.15 | 86.75 |
| 68 | BQ68 | 7.22 | 704.16 | 40.29 | 92.78 | 82.64 |
| 71 | BQ71 | 8.12 | 789.11 | 40.4 | 92.23 | 81.57 |
| 72 | BQ72 | 5.69 | 558 | 40.65 | 91.95 | 81.09 |
| 73 | BQ73 | 5.32 | 518.57 | 40.59 | 91.85 | 80.94 |
| 75 | BQ75 | 9.24 | 887.36 | 40.18 | 92.1 | 81.63 |
| 76 | BQ76 | 6.98 | 677.6 | 40.56 | 92.17 | 81.54 |
| 79 | BQ79 | 8.79 | 852.29 | 40.03 | 93.39 | 83.74 |
| 80 | BQ80 | 5.98 | 583.04 | 40.9 | 94.22 | 86.86 |
| 81 | BQ81 | 6.58 | 631.97 | 40.2 | 91.74 | 80.73 |
| 82 | BQ82 | 7.7 | 747.33 | 39.88 | 93.65 | 84.11 |
| 83 | BQ83 | 6.03 | 578.9 | 40.19 | 92 | 81.1 |
| 85 | BQ85 | 7.2 | 702.41 | 40.16 | 93.53 | 83.99 |
| 87 | BQ87 | 5.25 | 508.92 | 40.58 | 92.62 | 82.89 |
| 88 | BQ88 | 6.52 | 632.9 | 40.52 | 92.73 | 83.03 |
| 89 | BQ89 | 8.64 | 833.35 | 39.97 | 92.04 | 80.88 |
| 90 | BQ90 | 7.11 | 682.54 | 40.03 | 93.09 | 83.33 |
| 91 | BQ91 | 6.68 | 641.04 | 40.21 | 93.57 | 84.1 |
| 94 | BQ94 | 7.02 | 688.1 | 39.87 | 93.13 | 83.32 |
| 99 | BQ99 | 8.18 | 789.05 | 40.39 | 91.92 | 80.74 |
| 100 | BQ100 | 8.87 | 869.37 | 40.3 | 92.74 | 83.11 |
| 101 | BQ101 | 6.43 | 626.53 | 40.37 | 92.6 | 82.95 |
| 103 | BQ103 | 8.69 | 834.15 | 40.31 | 92.66 | 82.62 |
| 104 | BQ104 | 8.45 | 823.53 | 40.33 | 92.89 | 83.39 |
| 105 | BQ105 | 8.25 | 791.96 | 40.32 | 93.16 | 83.4 |
| 109 | BQ109 | 8.61 | 834.93 | 39.84 | 93.39 | 83.8 |
| 110 | BQ110 | 8.51 | 820.74 | 39.94 | 91.48 | 80.54 |
| 111 | BQ111 | 4.84 | 464.81 | 40.3 | 92.55 | 82.79 |
| 112 | BQ112 | 6.16 | 597.96 | 40.11 | 93.45 | 83.83 |
| 114 | BQ114 | 8.22 | 806.01 | 40.5 | 93.19 | 83.46 |
| 115 | BQ115 | 7.08 | 690.01 | 40.31 | 93.48 | 83.97 |
| 116 | BQ116 | 5.5 | 527.89 | 40.35 | 92.71 | 83 |
| 117 | BQ117 | 4.34 | 420.67 | 40.8 | 92.63 | 82.68 |
| 118 | BQ118 | 10.23 | 997.25 | 39.76 | 93.71 | 84.4 |
| 119 | BQ119 | 6.08 | 589.92 | 40.63 | 92.79 | 82.88 |
| 123 | BQ123 | 8.65 | 830.19 | 40.37 | 93.48 | 84 |
| 124 | BQ124 | 6.02 | 583.98 | 41.26 | 93.06 | 83.2 |
| 125 | BQ125 | 7.16 | 701.85 | 40.85 | 92.73 | 82.86 |
| 126 | BQ126 | 5.71 | 556.52 | 40.26 | 92.63 | 82.7 |
| 127 | BQ127 | 13.81 | 1332.22 | 40.84 | 94.43 | 87.32 |
| 128 | BQ128 | 5.63 | 546.18 | 41.44 | 92.98 | 82.98 |
| 129 | BQ129 | 12.07 | 1158.41 | 40.64 | 94 | 86.23 |
| 130 | BQ130 | 6.41 | 628.17 | 42.33 | 92.75 | 82.67 |
| 135 | BQ135 | 6.85 | 668.28 | 40.9 | 92.7 | 82.83 |
| 136 | BQ136 | 7.14 | 695.71 | 41.61 | 93.2 | 83.43 |
| 139 | BQ139 | 5.48 | 528.78 | 41.54 | 91.6 | 80.17 |
| 140 | BQ140 | 8.07 | 778.36 | 40.65 | 91.34 | 80.07 |
| 141 | BQ141 | 6.3 | 604.39 | 40.54 | 92.47 | 82.42 |
| 143 | BQ143 | 3.8 | 364.89 | 42.05 | 92.83 | 82.85 |
| 144 | BQ144 | 4.91 | 471.56 | 41.66 | 94.44 | 87.23 |
| 145 | BQ145 | 12.9 | 1251.39 | 41 | 94.5 | 87.43 |
| 147 | BQ147 | 6.61 | 634.78 | 40.65 | 92.83 | 82.97 |
| 149 | BQ149 | 6.91 | 670.2 | 41.64 | 93.19 | 83.41 |
| 150 | BQ150 | 7.61 | 738.13 | 41.28 | 93.34 | 83.6 |
| 152 | BQ152 | 7.32 | 710.15 | 40.78 | 94.23 | 86.86 |
| 153 | BQ153 | 5.13 | 502.32 | 41.86 | 93.17 | 83.4 |
| 155 | BQ155 | 9.18 | 895.51 | 41.29 | 93.57 | 84.1 |
| 157 | BQ157 | 4.5 | 436.4 | 40.94 | 94.3 | 87.07 |
| 158 | BQ158 | 16.09 | 1577.27 | 40.92 | 94.43 | 87.31 |
| 159 | BQ159 | 5.76 | 558.52 | 40.88 | 92.87 | 83.12 |
| 161 | BQ161 | 8.67 | 836.29 | 40.75 | 94.23 | 86.87 |
| 162 | BQ162 | 6.5 | 624.07 | 41.1 | 93.78 | 85.73 |
| 163 | BQ163 | 6.63 | 639.76 | 41.6 | 91.86 | 80.64 |
| 166 | BQ166 | 7.32 | 710.47 | 40.73 | 93.05 | 83.36 |
| 167 | BQ167 | 6.64 | 637.85 | 41.84 | 92.88 | 83.02 |
| 170 | BQ170 | 6.5 | 636.59 | 40.88 | 93.08 | 83.5 |
| 172 | BQ172 | 8.41 | 807.81 | 41.77 | 94.28 | 86.87 |
| 173 | BQ173 | 8 | 776.11 | 40.7 | 94.13 | 86.69 |
| 176 | BQ176 | 7.03 | 681.64 | 40.54 | 93.3 | 83.52 |
| 178 | BQ178 | 6.19 | 603.19 | 40.27 | 92.97 | 83.33 |
| 179 | BQ179 | 10.2 | 989.47 | 39.85 | 93.45 | 83.71 |
| 180 | BQ180 | 9.44 | 920.07 | 40.7 | 94.4 | 87.25 |
| 181 | BQ181 | 7.49 | 730.21 | 40.45 | 93.06 | 83.5 |
| 182 | BQ182 | 5.1 | 499.46 | 40.42 | 93.18 | 83.34 |
| 183 | BQ183 | 8.4 | 810.48 | 40.41 | 91.69 | 80.63 |
| 184 | BQ184 | 3.03 | 295.61 | 40.78 | 93.26 | 83.47 |
| 185 | BQ185 | 7.1 | 681.57 | 40.85 | 92.85 | 83.08 |
| 187 | BQ187 | 5.92 | 574.08 | 41.59 | 92.75 | 83.01 |
| 188 | BQ188 | 5.71 | 554.09 | 41.54 | 92.74 | 83 |
| 189 | 02428 | 12.32 | 1204.38 | 41.5 | 92.83 | 83.19 |
| 190 | Changhui 891 | 15.51 | 1492.63 | 41.79 | 93 | 83.33 |
